# How do people living with dementia perceive eating and drinking difficulties? A qualitative study

**DOI:** 10.1093/ageing/afab108

**Published:** 2021-06-11

**Authors:** Kanthee Anantapong, Yolanda Barrado-Martín, Pushpa Nair, Greta Rait, Christina H Smith, Kirsten J Moore, Jill Manthorpe, Elizabeth L Sampson, Nathan Davies

**Affiliations:** Marie Curie Palliative Care Research Department, Division of Psychiatry, University College London, London, UK; Department of Psychiatry, Faculty of Medicine, Prince of Songkla University, Hat Yai, Thailand; Centre for Ageing Population Studies, Research Department of Primary Care and Population Health, University College London, London, UK; Centre for Ageing Population Studies, Research Department of Primary Care and Population Health, University College London, London, UK; Centre for Ageing Population Studies, Research Department of Primary Care and Population Health, University College London, London, UK; Language and Cognition, Division of Psychology and Language Sciences, University College London, London, UK; Marie Curie Palliative Care Research Department, Division of Psychiatry, University College London, London, UK; National Ageing Research Institute, Parkville, Victoria, Australia; NIHR Policy Research Unit in Health & Social Care Workforce and NIHR Applied Research Collaborative (ARC) South London, King's College London, London, UK; Marie Curie Palliative Care Research Department, Division of Psychiatry, University College London, London, UK; Barnet Enfield and Haringey Mental Health Trust Liaison Team, North Middlesex University Hospital, Sterling Way, London, UK; Marie Curie Palliative Care Research Department, Division of Psychiatry, University College London, London, UK; Centre for Ageing Population Studies, Research Department of Primary Care and Population Health, University College London, London, UK

**Keywords:** dementia, nutrition, hydration, patient-centred, qualitative research, older people

## Abstract

**Background:**

Eating and drinking problems are common among people living with later-stage dementia, yet few studies have explored their perspectives.

**Objective:**

This study aimed to explore how people living with mild dementia understand possible future eating and drinking problems and their perspectives on assistance.

**Design:**

Qualitative study using semi-structured interviews.

**Setting:**

Community.

**Methods:**

We conducted semi-structured interviews with 19 people living with mild dementia. Interviews were transcribed verbatim and analysed thematically.

**Results:**

Five themes were identified: (i) awareness of eating and drinking problems; (ii) food and drink representing an individual’s identity and agency; (iii) delegating later decisions about eating and drinking to family carers; (iv) acceptability of eating and drinking options; and (v) eating and drinking towards the end of life. For people living with mild dementia, possible later eating and drinking problems could feel irrelevant and action may be postponed until they occur. Fears of being a burden to family and of being treated like a child may explain reluctance to discuss such future problems. People living with mild dementia might wish to preserve their agency and maintain good quality of life, rather than be kept alive at later stages by artificial nutrition and hydration.

**Conclusion:**

For people with mild dementia, eating and drinking problems may seem unrelated to them and so get left undiscussed. Negative connotations regarding eating and drinking problems may hinder the discussion. The optimal time to discuss possible future problems with eating and drinking with people with mild dementia may need an individual approach.

## Key Points

- The likelihood of future eating and drinking problems is often not recognised by people in the early stage of dementia.
- People living with mild dementia may want to delay discussion of possible future eating and drinking problems.
- Fears of infantilisation and being burdensome may hinder people living with dementia discussing possible problems.
- People living with dementia often accept that they will gradually delegate the decision-making to family carers and professionals.
- Nutrition and hydration interventions for people with advanced dementia should aim at maintaining dignity and quality of life.

## Background

People living with dementia experience progressive cognitive decline, behavioural changes and motor disturbances. This commonly results in eating and drinking problems as symptoms progress. Over 80% of people with dementia encounter at least one eating and drinking difficulty, such as swallowing problems, loss of appetite, inability to recognise food and utensils, difficulties in attention and problems maintaining an eating routine [1]. These may affect physical and psychological well-being. Decreased oral intake in the advanced stages might not cause discomfort for people living with dementia [2–4]. However, family carers and professionals may feel obligated to continue feeding to avoid feeling they are being neglectful [5–7]. Eating and drinking difficulties may also affect relationships between the person with dementia and others [8, 9].

People living with dementia who develop eating and drinking problems can be supported by encouragement, food texture modification and fluid thickener, adapted utensils, environmental adaptation, and careful hand feeding [10, 11]. People with subtle eating and drinking problems at the mild stage can be supported to continue oral eating and drinking with some of these adjustments; however, support specific to this population is less clearly outlined in most guidelines [12–14]. This could lead to possible eating and drinking problems from dementia not being discussed in the earlier stages of dementia. Invasive ways such as Artificial Nutrition and Hydration (ANH) may be used when other measures such as encouragement and food texture modification seem no longer effective, especially at the more severe stages of dementia [15]. However, the use of ANH for people living with dementia does not improve quality of life, nutritional status, prevent recurrent aspiration or prolong life [16, 17]. Recommendations for dementia care towards the end-of-life focus on the person’s well-being and on avoiding the overuse of life-sustaining and invasive interventions [12, 18].

Most studies on eating and drinking for people living with dementia have focussed on family carers and professionals, or analysed routinely collected data about people living with dementia. Little is known of the perspectives of people with dementia themselves about eating and drinking problems [15, 19]. Hence, we aimed to explore the experiences, needs, and opinions of people living with mild dementia about possible future eating and drinking problems. Our specific research questions were:

1. How do people living with mild dementia understand eating and drinking difficulties that may occur in the later stages of dementia?
2. How would people living with mild dementia like future eating and drinking decisions to be made?
3. What strategies to support future eating and drinking are acceptable to people living with mild dementia?

## Methods

### Design

We designed a qualitative study using semi-structured interviews. Interviews were analysed using thematic analysis.

### Data collection methods

During September 2019 to March 2020, two researchers (YBM, PN) with backgrounds in psychology and medicine, conducted face-to-face semi-structured interviews with people with mild dementia. Each interview lasted approximately 1 hour and was guided by an interview schedule that was developed in consultation with stakeholders including a Patient and Public Involvement (PPI) group, and professionals working in dementia care, palliative care, and speech and language therapy. The interview schedule contained questions about current eating and drinking difficulties, anticipated problems in later stages, the individual significance of eating and drinking, decisions around eating and drinking problems, and possible strategies. To facilitate discussions, we presented vignettes that described a fictional person’s eating and drinking problems across the dementia trajectory (see Appendix 1). Interviews were audio-recorded, pseudonymised and transcribed verbatim. We conducted individual interviews of people living with dementia but gave the option of being interviewed with a family member if preferred.

### Eligibility

We screened potential participants by using eligibility criteria (Table 1).

**Table 1:** Eligibility criteria of participants

Inclusion criteria
- Clinical diagnosis of any type of dementia as categorised in ICD-10^*^
- Mental capacity to provide informed consent
- Participants must be able to read and speak English
Exclusion criteria
- Where there are clinical or social concerns that preclude them being approached

^^*^^ICD-10 = International Statistical Classification of Diseases and Related Health Problems 10th Revision.

Two trained researchers (YBM, PN) performed the assessment of potential participants’ ability to understand, retain and use or weigh-up information, and communicate their decision, guided by the UK Mental Capacity Act (2005). If they had capacity to consent for this study, we then obtained written informed consent from them.

### Sampling and participant recruitment

We purposively sampled participants with mild dementia in Greater London and South-East England. We recruited for age, gender, ethnicity, religion and location to maximise diversity.

Participants were recruited from National Health Service (NHS) memory services, primary care practices, Join Dementia Research (JDR), not-for-profit dementia organisations and online social media. Clinical staff in memory services and primary care practices identified and approached potential participants by providing them an information sheet. The research team also sent the information sheet to potential participants who registered with JDR and the inviting organisations. The research team then contacted the participants, who had returned an expression of interest to take part, using their preferred form of communication.

### Analysis

Interview transcripts were uploaded to NVivo 11 and analysed using thematic analysis methods led by KA with team-based discussions, to enhance rigour [20]. Two researchers (KA, YBM) independently coded two interviews and developed a coding framework that was finalised and agreed in discussions among three researchers (KA, YBM, ND). The remaining interviews were coded by KA, and regularly discussed with YBM and ND. The emerging themes were developed and refined through iterative discussions among all researchers and feedback from PPI members.

### Reflexivity

The research team consisted of psychologists, old age psychiatrists, general practitioners, social care researchers, and speech and language therapists working with people with dementia and carers and with experience of family dementia care. Our research focuses on dementia care at the end of life, and we share a general view that eating and drinking at this stage should focus on quality of life and comfort rather than maintenance of nutritional status. This might have influenced the research. We reflected on our views throughout the research and considered their impact. We also included PPI members to help us design the study and interpret the data to strengthen our approach.

### Ethical consideration

Ethical approval was granted by Health Research Authority (HRA) committee of England (Queen Square Research Ethics Committee; REC reference: 19/LO/0369).

## Results

### Participants

Characteristics of the participants in this study are summarised in Table 2.

**Table 2:** Participant characteristics (*N* = 19)

Characteristics	*N* (%)
Age (years)	
Mean (SD)	76.7 (8.0)
Median	79
Range (Min, Max)	57,88
Gender	
Female	9 (47.4)
Male	10 (52.6)
Marital status	
Married or in a civil partnership	11 (57.9)
Widowed	3 (15.8)
Divorced	3 (15.8)
Separated	1 (5.3)
Single	1 (5.3)
Ethnicity	
British, Irish or White other	14 (73.7)
Indian/Asian	3 (15.8)
African/Caribbean	1 (5.3)
Other ethnic group	1 (5.3)
Education completed	
<15 years	3 (15.8)
15–16 years	5 (26.3)
17–20 years	3 (15.8)
21+ years	8 (42.1)
Dementia subtype	
Alzheimer’s disease	8 (42.1)
Vascular dementia	3 (15.8)
Frontal lobe dementia	2 (10.5)
Lewy Body Dementia	1 (5.3)
Parkinson’s Dementia	1 (5.3)
Mixed dementia	3 (15.8)
Unsure	1 (5.3)
Time from diagnosis (years), mean (SD)	2.1 (1.8)

### Key findings

Many people living with mild dementia interviewed (henceforth participants) were not aware that eating and drinking problems frequently emerge as part of dementia. They seemed happy to engage in discussion about them during the interview but did not feel it was relevant to continue this discussion beyond the interview and as part of planning for their own care. They preferred to leave such discussions and decisions to family carers and professionals when problems occurred. In their view, eating and drinking strongly affected their quality of life and sense of identity. Hence, there was limited acceptability of the use of eating and drinking interventions, especially at the end of life. We categorised and present the findings into five overarching themes as follows.

### Awareness of eating and drinking problems

Participants generally discussed dementia symptoms and overall progression in relation to memory problems and decreasing levels of awareness. Eating and drinking problems were not yet considered relevant by some who had not experienced them (see illustrative quote ‘Theme number 1 Quote number 1’ (T1Q1) in Table 3). Many participants focused on the present and hoped that they would die before developing such problems (T1Q2). A few felt the whole subject was irrelevant to them and avoided discussing it.

**Table 3:** Illustrative quotes from people with mild dementia

Theme 1: Awareness of eating and drinking problems	
T1Q1	The bigger problem to me is thinking and not being able to remember things. Forgetting people’s names and really think around in your mind and never get it, and suddenly out of the blue, 2 min later, it comes into your mind. To me, that’s the area of problem, nothing to do with eating or anything. (P12, male, 81–85 years old)
T1Q2	But to be honest, I don’t think that we will arrive there (having eating and drinking problems). I will die before. I am 7X. (P16, male, 76–80 years old)
T1Q3	It’s later. I mean call me in a years’ time, and my life might have changed. And then we can talk more, if I can still talk. And probably you would involve my wife because she would be the carer. (being asked about a discussion on eating and drinking problems) (P02, male, 76–80 years old)
T1Q4	If they understand. . .that everything’s going to be degraded at. . .the end stage. Then these questions would not be so troubling for them and so they could be handled in advance. (being asked about advance discussions on eating and drinking problems) (P17, male, 81–85 years old)
Theme 2: Food and drink representing an individual’s identity and agency	
T2Q1	I don’t know if it’s good to say, but I think this illness has changed me completely. That’s why I have this faith that I’m going to be better, because I’m doing all the right things and it is that has changed me to live the way I am. (referring to eating healthy food) (P15, female, 71–75 years old)
T2Q2	I’m sorry that my difficulties with swallowing bar me from joining in the food eating in company because I feel that’s a shame that I’m missing out on that, but I just can’t bear to be where people are shovelling. . .. I just didn’t want to be there when all I could eat was grapes. (P09, female, 71–75 years old)
T2Q3	They’d be changing, reversing it round. I’m the mother of them and then it would turn around and they’d be the mother of me almost, and I wouldn’t like that. (P03, female, 76–80 years old)
T2Q4	But if the person is in really advanced dementia, I’m not sure how much they’ll notice that and if they do notice it wouldn’t really be a problem to them. They’re not active. I don’t imagine they’re active; do they get up and walk around? (being asked about gastrostomy and intravenous hydration) (P17, male, 81–85 years old)
Theme 3: Delegating later decisions about eating and drinking to family carers	
T3Q1	I wouldn’t really know how I could make a decision. . .So, I would depend on my carer to do what they thought was the right thing to do. . .I don’t really want to know. All I really want to make sure is that the carer doesn’t have a problem, and that my wife can sort of make the right decisions, for her. (P02, male, 76–80 years old)
T3Q2	If you’re under someone’s supervision, they’re more knowledgeable about these things presumably, and there are other people in the same condition, in the same place where you’re living, maybe that would encourage someone to eat a bit more. (P04, female, 76–80 years old)
T3Q3	I think the family should work with the doctors. It’s team work, family, doctors, psychiatrists and everybody else involved in this field, and come up with what’s best for the individual. . .Because even me myself won’t be in a position to make a proper decision. (P15, female, 71–75 years old)
T3Q4	It’s difficult, isn’t it? I think force feeding, you know what I mean, I don’t think that’s a very good idea. If the person is asking for food, then I think they ought to be given it, as long as it’s not something that is obviously going to be harmful. But I think if people choose not to eat, I think it’s their right. (P02, male, 76–80 years old)
Theme 4: Acceptability of eating and drinking options	
T4Q1	I have heard about it obviously, but the thought of having a drip feed. . .They have it through their nose and things like that. Oh, I can’t bear the thought of it, but then. . .I suppose you should do it to keep somebody alive, but I wouldn’t want it done. You know what I mean? I’m looking at it from two angles really. (P03, female, 76–80 years old)
T4Q2	I think if that’s the only way that you can be fed, you can’t let a person starve. . .To be honest, I think if I refused food my family would be very, it would be hard for them and I would probably feel that I don’t want to put them through that. (being asked about the use of tube feeding at the end of life) (P20, female, 56–60 years old)
T4Q3	I would imagine if I refused to eat. . .it’s one thing to be coaxed into it, it’s another thing to be pushed into it, because then you get nervous and you push back and then you begin to look upon the next meal as a battle. And then you begin to fear the next meal, and they come three times a day or minimum twice. (P17, male, 81–85 years old)
T4Q4	Oh, hydration is really paramount. You can do without food for a while but you can’t do without water for very long. . .It’s more immediate. (P17, male, 81–85 years old)
Theme 5: Eating and drinking towards the end of life	
T5Q1	I have no idea, I would imagine at some point they probably don’t even feel hunger, they don’t notice it if they do; the body will notice it but consciously they may not. (being asked about level of hunger at the end of life) (P17, male, 81–85 years old)
T5Q2	Well, you can’t keep offering food to people who don’t want to eat it, can you? I don’t quite know what the situation would be, but if it were me and I didn’t want to eat and I’ve decided that this was the end, then I want to be left alone and kept comfortable. (P04, female, 76–80 years old)
T5Q3	I don’t want these lining-up tubes top to head. You’re not going to get any better. You are better for a week or two, but it won’t be long. (P15, female, 71–75 years old)
T5Q4	I think that Mrs S (in vignettes) is actually getting to a stage in her life when she’s started to disengage from life, and I think that that should be allowed to happen, with kindness and support. . .I think she (daughter of Mrs S) might want to encourage her mother to eat, but I don’t think she should do anything more than that. She should respect her mother’s wish not to eat, because I think it’s part of the preparation for dying. (P09, female, 71–75 years old)

Those mentioning eating and drinking matters spoke more about mild changes in their appetite and preferences, and less about physiological changes. For example, participants spoke of wanting less food, preferring healthier diets and cooking for themselves more often instead of eating out. Some reported feelings of dryness or discomfort in their throat, although some termed these swallowing difficulties after being asked about swallowing difficulties. Most participants did not acknowledge eating and drinking difficulties until asked about changes in their eating and drinking; and many did not attribute these to dementia. In their view, such changes were a result of their own choices or due to other comorbidities, including dental problems, diabetes and Parkinson’s disease. Changing life circumstances, such as retirement, moving house, financial crisis and caring for relatives, was described as limiting their ability to eat out and socialise.

Participants stated a preference for leaving the subject of eating and drinking difficulties until problems arose. Some acknowledged, however, that people with dementia have less awareness or ability to discuss the problems at the later stages (T1Q3). Most participants could confidently explain whether they currently had eating and drinking difficulties and what kind. They also clearly expressed a view about what should be done with regards to eating and drinking at the end of life, which they hoped would be mainly aimed at keeping them comfortable and free from pain, being surrounded by people whom they loved, and dying peacefully. There was little discussion of possible physiological problems across the dementia progression, especially at moderate or severe stages. Few participants wished to know more about eating and drinking problems at the later stages (T1Q4).

### Food and drink representing an individual’s identity and agency

For participants, the ability to eat and drink represented quality of life, sense of identity and agency. At the time of interviews, eating and drinking was one means of ‘staying healthy’ and so represented something that they could control. Many hoped that if they controlled their diets at the early stage, it would prevent further decline (T2Q1). For some, weight loss was not a problem because they had always tried to lose weight and preferred to be slimmer. Some preferred to prepare food for themselves, and sometimes for others. Food preparation helped maintain their freedom to choose ingredients, flavours, nutrients and calories. These were suggested as a way to ensure that their identity and agency remained respected. Although most participants perceived that having meals with others generally helped encourage eating and drinking, some expressed unease. They acknowledged tending to avoid eating with other people because of difficulties with swallowing and using utensils, while a few reported food preference changes that might be noticed (T2Q2). Some participants felt that others might be excluding them gently because of their difficulties with eating and drinking.

Most participants were uncertain about what their health would be like and what eating and drinking problems they would have at later stages. They envisaged having less control over eating and drinking activities in the future. Most thought they would be gradually transferring responsibility to family carers, despite their desire to maintain agency for as long as possible. When talking through the case vignettes some were able to discuss specific situations in the later stages of dementia. In cases of refusing to eat or drink, many participants mentioned techniques that are traditionally used with children to encourage eating and drinking, for example, giving rewards, coaxing, and doing ‘aeroplane’ (i.e. pretending the spoon of food is an aeroplane). Most did not like these techniques as it represented role reversal and being treated like a child; undermining their identity (T2Q3). However, some supposed it might be the only way people could help them despite acknowledging that it would not be ideal. There was general opposition to ANH as they perceived that it was unnatural and would not bring them enjoyment and quality of life. For a few, being at the very advanced stage of dementia was one where they envisaged they would probably not mind or feel distressed from what people were offering them as they would have less understanding and awareness of their situation and surroundings (T2Q4).

### Delegating later decisions about eating and drinking to family carers

Many participants understood that they would gradually lose memory and decisional capacity. Some assumed that at the later stages they would not be able to make decisions regarding eating and drinking. They would like to leave decisions to family carers because they trusted them to consider their own well-being and ability to provide such care (T3Q1). However, they were concerned about burdening family carers and restricting their normal life. Some considered that a move to a care home or hospice would be fair to their families, and then they would receive professional care (T3Q2). Nonetheless, home was described as the best place to live and die.

Participants hoped healthcare professionals would explain and help their family carers with any problems related to eating and drinking at the right moment, for example, if being treated with an acute transient illness or approaching the end of life. In their views, professionals were best placed to know about risks and benefits (T3Q3). Professionals should share such information with family carers. When participants discussed the vignettes, many found it difficult to see themselves in those situations. However, participants often referred to the experiences of other family members or friends with dementia or other terminal illnesses who needed support with eating and drinking, including feeding and ANH. These had given familiarity with some feeding interventions and influenced their views. For example, some would agree with food texture modification, risk feeding, or even ANH if they had seen it was useful for other people with or without dementia. Participants who were more accepting of ANH were those who did not really understand the possible eating problems and interventions. However, participants still looked to their family carers to respect their wishes, especially if they were wanting certain food and drink or were strongly refusing any food and drink (T3Q4).

### Acceptability of eating and drinking options

Participants felt they would generally accept encouragement to eat and drink by either carers or professionals if they were having eating and drinking problems in the later stages. They wanted people to offer them the food they enjoyed or requested at the time, to encourage them to eat and be around to help if needed. They felt that they would be willing to try food and drink with texture modification, such as pureed food and sparkling water. Small amounts of alcohol were thought a possible way to encourage hydration; however, many participants had already stopped drinking alcohol for health reasons.

At the later stages, ANH was often perceived as making someone uncomfortable and keeping them alive unnecessarily. Some participants thought that ANH might be acceptable to keep other people with dementia alive, but not for themselves (T4Q1). Very few people living with dementia viewed the use of ANH as appropriate. For those who did, it seemed to be because they thought it would be the only option for avoiding death by starvation, which could be distressing for their family (T4Q2), but again this was not what they would want for themselves. While family carers and professionals could encourage them to eat and drink, this definitely should not be against their wishes. Some were concerned about possible ‘battles’ between themselves and others around meals, which would lead to worry about subsequent meals (T4Q3). If an individual strongly refused to eat, they felt that family carers should stop feeding and just keep them warm and comfortable.

Despite the reluctance to have artificial nutrition, participants felt that in the very late stages of dementia the practice of maintaining hydration by offering drinks and artificial hydration like intravenous fluids was more acceptable. This was because hydration was perceived to have more immediate effect on the quality of both life and death of people with dementia (T4Q4).

### Eating and drinking towards the end of life

Participants were unsure whether their appetite would completely disappear at the end of life, or if they would still be hungry (T5Q1). Some participants were concerned about the risk of choking if food was offered and would prefer to be offered sips of water or left alone. For some who discussed a possible scenario of having an acute illness, ANH, particularly intravenous fluids, seemed acceptable because they did not want to die prematurely and thought there might be no other option. Nonetheless, if they resisted eating and drinking, or the use of ANH at the time, participants thought this could mean a person did not want to stay alive (T5Q2).

Some participants had thought about euthanasia before they reached very advanced dementia but considered it impossible in the present legal context. If approaching death, most participants did not want ANH especially tube feeding. Many participants mentioned that they would not be afraid of naturally dying, but of living in a poor condition, being no longer themselves, and being a burden to their family. In this case, careful hand feeding was a preferred mode of taking food. Some thought they would request stopping feeding, as they perceived they would be close to death (T5Q3). All preferred to stay comfortable and be respected while they were still living and ‘to fade away’ peacefully when death was approaching (T5Q4).

## Discussion

This study brings new perspectives on eating and drinking difficulties in people living with dementia. It is the first study reporting direct experiences, future wishes, and the opinions of people with mild dementia on this topic. Generally, participants recognised some mild changes in eating and drinking but did not attribute the changes to dementia. They also were not aware of eating and drinking difficulties associated with the later stages. Eating and drinking were part of their sense of identity and agency, which they wished to protect for as long as possible. However, they acknowledged the progressive trajectory of dementia and felt that they would defer decision-making to family carers to help preserve their quality of life, especially at its end.

### Awareness and perception of eating and drinking problems

Participants generally did not recognise the likelihood and seriousness of having eating and drinking problems in the future. This may delay their acknowledgement of eating and drinking difficulties in the mild stages of dementia and possibly hinder them from talking to carers and professionals about their preferences and decisions should such difficulties about eating and drinking progress. Carers and professionals themselves often find it difficult to know the preferences of the person with dementia for care decisions at the later stages [6, 21, 22]. Most participants preferred to leave discussions until difficulties arose, despite knowing that they might have less decisional capacity to engage later. While there is much encouragement of people living with dementia participating in healthcare decision-making [23, 24], it seems people do not always wish to do this and may be unduly optimistic that they will be unaffected by eating and drinking problems [23, 25, 26].

Participants generally focused on their present eating and drinking ability and ‘minor’ changes at the mild stage. Eating and drinking were considered fundamental to the participants’ health and quality of life, and to bring them enjoyment, and sense of agency and social inclusion. They seemed able to adjust to the minor changes and sometimes normalised the adjustments to their eating and drinking patterns as an attempt to delay dementia progression. Unlike participants in this study, older people without cognitive impairment but at risk of malnutrition and dehydration might not always be concerned to have healthy, adequate diets and avoid alcohol consumption to slow or prevent certain illness [27, 28].

Although this could have been led by our interview schedule, participants could outline what they wanted right at the end of life but seemed not to be aware of the risks of eating and drinking difficulties at moderate and severe stages, i.e. ‘in between the present and the end’ such as commonly encountered difficulties with swallowing, recognising food, using utensils and behavioural changes [1]. Difficulties in the later stages may be over-looked at their earliest manifestations since they could give rise to feelings of being treated like a child and needing more help [1, 9, 29, 30]. It is difficult for others to introduce discussions on eating and drinking problems to people with mild dementia who have limited or mixed awareness of the problems because this can result in frustration, embarrassment and shame [31]. However, we found that when we gradually introduced possible eating and drinking problems and used vignettes, participants could engage more with the subject. This seemed an effective way to elicit their values and views about topics that may not appear currently relevant.

In both primary and secondary healthcare, there is variation, in terms of amount and timing, in how information about dementia care is relayed to people with dementia and carers [12]. Information regarding post-diagnostic support and end-of-life care can sometimes seem overwhelming. Professionals are recommended to regularly assess the person’s needs and discuss their care plan throughout the dementia progression [12]. Honest, sensitive, supportive and successive conversations and education, especially about moderate and severe stages, may help people living with dementia to access information when they are ready and develop realistic, personal plans [22, 25, 32].

### Interventions and decision-making about food and eating

Eating and drinking interventions that were favoured by participants included the provision of favourite foods and food texture modification. They thought eating with others might help, although some reported that eating and drinking difficulties were already precluding them from socialising, of which people living with dementia may feel embarrassed and in turn withdraw from social interactions further [31]. Such approaches to encourage eating have promising benefits in terms of improving nutritional status, quality of life and social engagement [10]. However, at the advanced stage of dementia, these interventions require time from carers supplemented by good homecare services [19, 30]. In line with fears of being treated like a child, notions of ‘role reversal’ of spousal and parent–child relationships may create anxiety in people with dementia and fears of being burdensome to family [9, 29, 32]. ANH was also deemed unacceptable by participants in this study; however, in previous studies some carers and professionals might perceive ANH was unavoidable, or want to try every available option [15, 19]. Participants stated wanting to take carers’ well-being and opinions into account, together with recommendations from professionals when making decisions about eating and drinking. This may suggest support for shared decision-making with input from people with dementia, family carers and health and care staff. Ideally, shared decision-making helps communication, enhances knowledge and understanding and improves satisfaction with care [15, 17, 24, 31].

As dementia progresses, people with mild dementia anticipated having to gradually delegate decision-making to carers and professionals. This is consistent with previous research reporting that over time carers needed to take charge of decisions but were still committed to engaging the person with dementia as far as they could, as part of a shared decision-making process [26, 33]. Participants seemed to have high levels of trust not only in their carer but also in professionals; this was evident across the ethnic diversity in our sample, but it may result from our participants having high levels of formal education [34]. Those who had high levels of education were largely recruited via online platforms as they might have more access to internet. While participants generally wanted to maintain their identity and sense of agency for as long as possible, previous studies found that people living with dementia would appreciate subtle support [23, 31]. Participants wished carers and professionals respecting their wishes to continue or pause eating and drinking. However, for carers and professionals, refusals to eat and drink by people with advanced dementia are often complicated and difficult to interpret [35]. Our findings suggest that what someone says or does in the moment may not last and offers of food can acceptably be made later. The main purpose of eating and drinking for people living with dementia may not be to maintain weight and have optimal nutrition, but to benefit from the psychosocial aspects of eating and drinking that retain their identity, autonomy and quality of life. At the end, participants saw it important that someone should die peacefully, with life not prolonged unnecessarily by ANH.

### Strengths and limitations

To our knowledge, this is the first study using a qualitative design to seek understanding of experiences and opinions about eating and drinking directly from people living with mild dementia. We interviewed a range of people living with mild dementia with maximised variation of participant characteristics. Data analysis involved multiple analysts with various backgrounds including PPI members, and iterative team-based discussions in developing codes and themes. These approaches enhanced the analytical rigour of the study.

We acknowledge that participants might not fully have understood the dementia progression and our questions. However, we tried to use open questions, reframed any questions to be easier to understand, and clarified unclear points. Eight participants were interviewed with carers who helped rephrase some questions to the participants and occasionally shared their opinions. This stimulated, rather than inhibited, the person with dementia to further discuss. However, on a few occasions a carer’s views could have led to the person changing their position. Introducing sensitive topics like eating and drinking difficulties could also have caused some psychological distress. However, research can help explore participants’ ideas on topics that are under-recognised resulting in new understandings of certain phenomena if sensitively conducted. The interviews were undertaken by experienced researchers, aware of the potential of distress with a protocol for following up anyone who might be distressed or wished to take the discussion further.

## Conclusion

For people with mild dementia, eating and drinking problems may seem irrelevant to them and so get left undiscussed until problems occur. Partly, fear of being a burden to family carers and of being treated like a child, inhibited discussion. Participants would accept some adjustments to eating and drinking problems at the mild stage, and may do this themselves. However, ANH, used at the later stages, was considered different because it was thought to be unnatural and threatened their autonomy. Most participants expressed a wish to preserve their agency and maintain good quality of life, rather than be kept alive unnecessary. Timely, successive discussion and psychoeducation with a sensitive approach to personal wishes may help people consider and plan for any future problems and should be part of the system of post-diagnostic support in dementia care.

## Supplementary Material

aa-20-1721-File001_afab108
