# Supplementary material for: How do people living with dementia perceive eating and drinking difficulties? A qualitative study
Source: Age Ageing. 2021 Jun 11;50(5):1820–8. doi: 10.1093/ageing/afab108 (PMC8522686; doi:10.1093/ageing/afab108)
Supplement: aa-20-1721-File001_afab108 [file aa-20-1721-file001_afab108.pdf]

Title: How do people living with dementia perceive eating and drinking difficulties? A qualitative study

Appendix 1: Case vignettes

We have some specific scenarios which we would like to discuss with you which you may not have mentioned. Please let me know if you would not like to continue at any point.

We wanted to understand how you would want your family or care team to approach some of these scenarios at home. *[Participants given each case study on a separate sheet to read and interviewer asks the questions below]*

<u>Vignette A</u> – Mrs S is 86 and has vascular dementia. She lives at home with her daughter and her family. Mrs S has recently started to eat less and less and no longer seems to find pleasure in foods she once really enjoyed. She often leaves food at the end of her meal and finds it more and more difficult to feed herself. At times Mrs S refuses to eat. Her daughter does not know what is the right thing to do and if she should encourage her mum to eat or not.

1) Would you want to be encouraged to eat?
    a. Why, if so?
    b. Why, if not?
    c. How could you be encouraged to eat, or eat more?
    d. What techniques would you want your family or someone to use?
    e. Are there particular food or drinks that they prefer and cause less problems?

<u>Vignette B</u> - Mrs S has continued to experience swallowing difficulties for some time and though careful thought in providing suitable foods for her meals has prevented further choking episodes, her daughter is having a lot of problems in giving her medication. It has been possible to substitute with liquid forms for many of her tablets and some have been dropped but the big issue is with her aspirin. Although a soluble form has been prescribed, the aspirin still leaves a gritty residue in the water, Mrs E is now refusing to take it as it has caused her to choke a couple of times recently. Her GP is very reluctant to stop it.

1) What would you want your family member to do in this instance?
    a. What should they consider?
    b. Does this depend on the type of medication?
    c. What would be your main concerns?
    d. Is home still the right place to stay at this time?

<u>Vignette C</u> – Mrs S has continued to find difficulties with eating. All her foods are now pureed and her daughter often uses sweet foods which she knows her mum enjoys. Her daughter has been spending time with her mum providing careful hand feeding. Liquid is thickened to ensure she does not choke. However, even careful

hand feeding no longer works. Mrs S does not swallow food and holds the food in her mouth. Her daughter is afraid she will choke and is not getting any nutrition. Mrs S health is becoming worse and the clinical team have said she is approaching the end of life.

1) What would you want your family member to do in this instance?
    a. What should they consider?
    b. Does this depend on the type of medication?
    c. What would be your main concerns?
    d. Would you want tube feeding to be used?
    e. How would you want to remain hydrated?
    f. What would you be happy with your family doing?
    g. Is home still the right place to stay at this time?
